# Supplementary material for: A Novel Rhodotorula evergladensis CXCN-6 Rich in Torularhodin and PUFAs with Potent Antioxidant and Anti-Inflammatory Activities
Source: Antioxidants (Basel). 2025 Nov 27;14(12):1420. doi: 10.3390/antiox14121420 (PMC12729354; doi:10.3390/antiox14121420)
Supplement: Supplementary file 1 [file antioxidants-14-01420-s001.zip › antioxidants-4003077-supplementary.pdf]

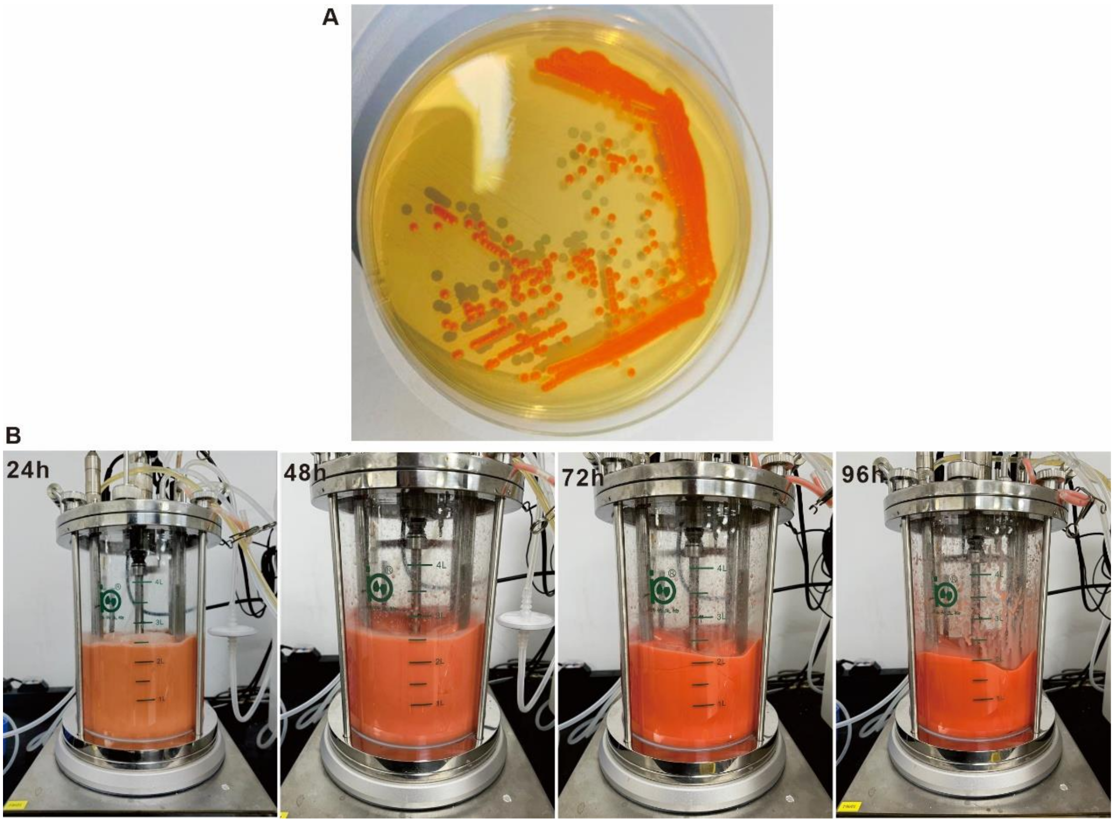

Figure S1. ***R. evergladensis*** **CXCN-6 strain exhibited an intense reddish-orange pigmentation in both liquid and solid YPD cultures. (A)** Colony morphology on YPD agar after 48 h at 28 °C showing smooth, glossy, and orange-red colonies. **(B)** Cell pellets from liquid culture displaying uniform reddish coloration indicative of intracellular carotenoid accumulation. The pronounced pigmentation reflects active carotenoid biosynthesis, predominantly torularhodin, within the yeast cells.

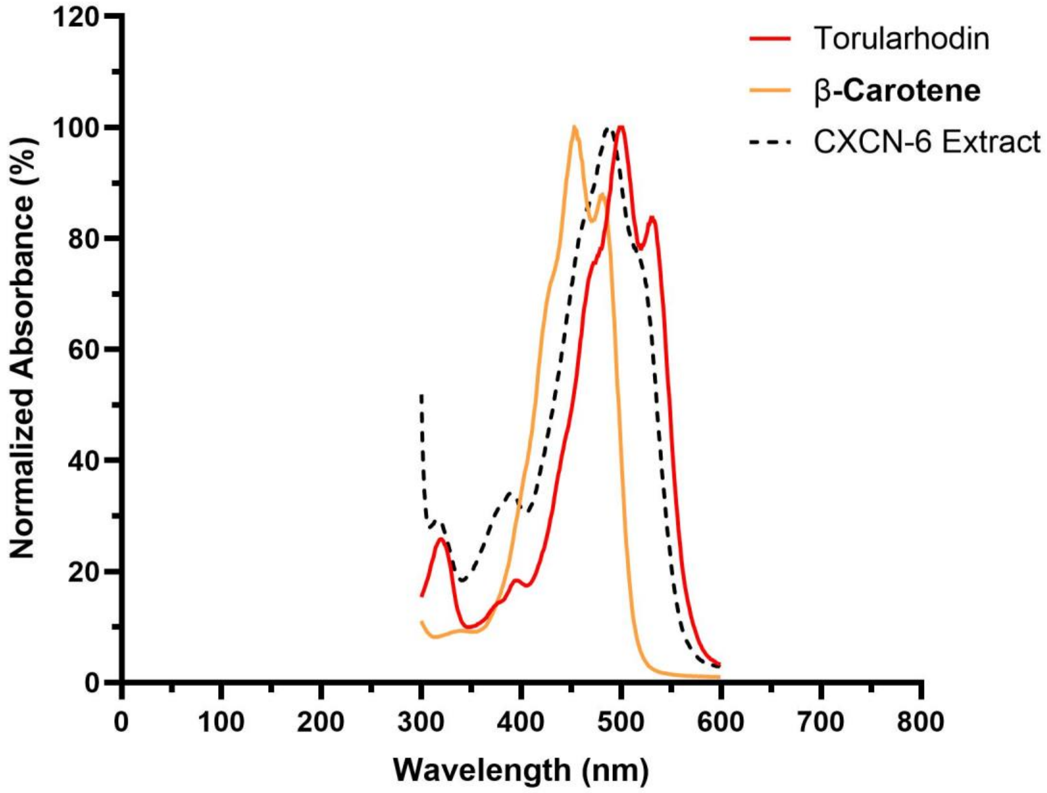

Figure S2. UV–Visible absorption spectra of torularhodin, β-carotene, and the ethanolic extract of *R. evergladensis* CXCN-6. The absorption spectra were recorded between 300 and 600 nm to characterize the pigment composition. The CXCN-6 extract exhibited a strong absorption maximum at approximately 490 nm, consistent with the characteristic peak of torularhodin. The spectral pattern closely matched that of the torularhodin standard, confirming that torularhodin is the predominant carotenoid component in the extract. The overall spectral intensity increased with cell growth, indicating progressive intracellular accumulation of carotenoids during fermentation.

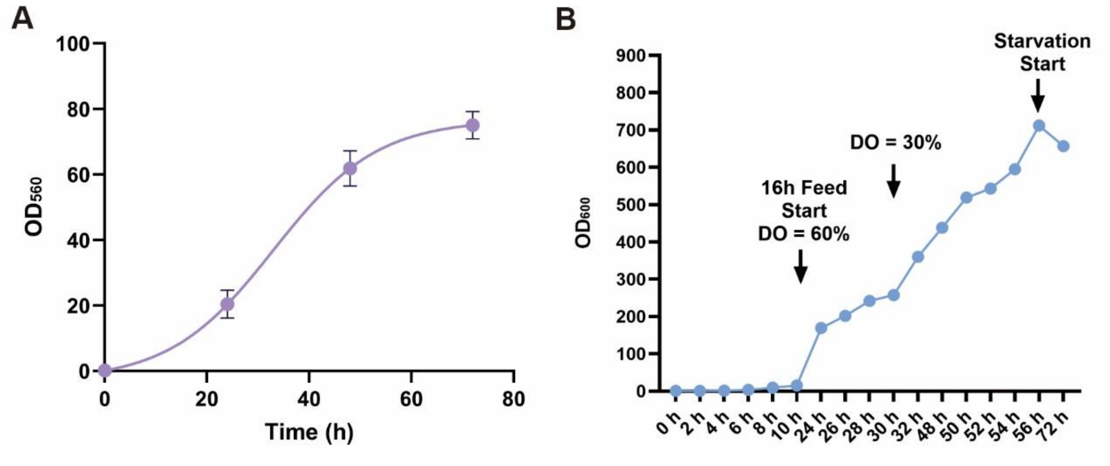

Figure S3. Growth kinetics and induction of secondary metabolite synthesis in *R. evergladensis* CXCN-6. (A) The growth curve revealed that biomass accumulation slowed markedly after 72 h, indicating entry into the stationary phase and a metabolic transition from primary growth to secondary metabolite production. **(B)** To stimulate intracellular carotenoid and lipid accumulation, a nutrient-limiting strategy was applied by terminating the feeding process at 56 h, inducing mild starvation stress known to enhance secondary metabolism in oleaginous yeasts.

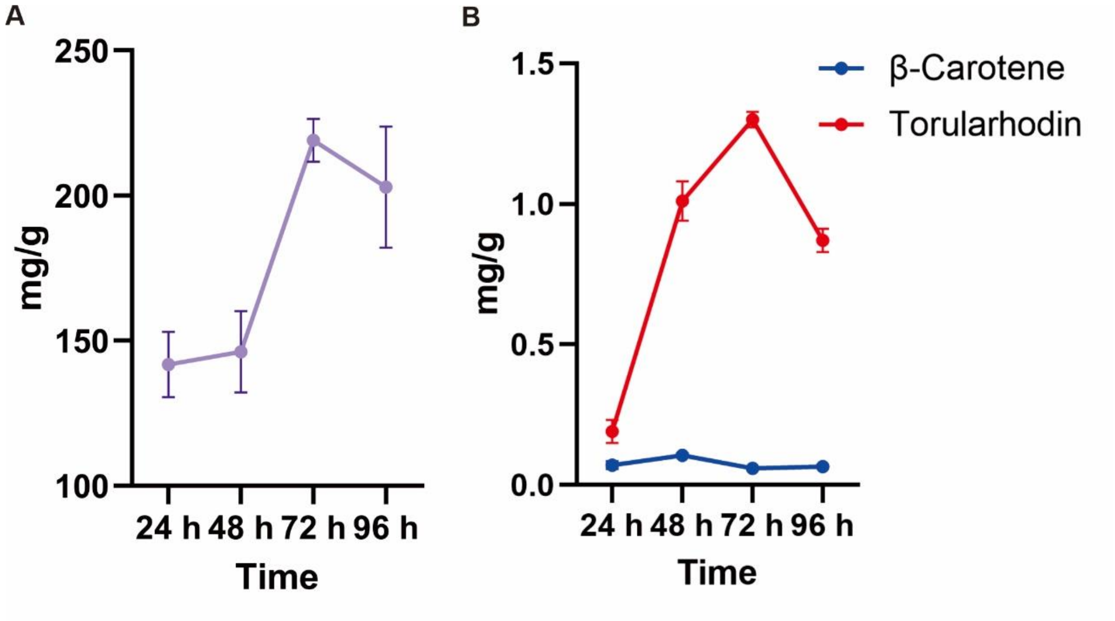

Figure S4. Time-course profiles of torularhodin and total lipid production in *R. evergladensis* CXCN-6 during fermentation. The changes in **(A)** total lipid content (mg/g DCW) **(B)** torularhodin content (mg/g DCW) were monitored at different time points during 5 L bioreactor fermentation. Both torularhodin/DCW and total lipids/DCW increased steadily during the late exponential and stationary phases, reaching their maximal levels at approximately 72 h. The accumulation patterns indicate a metabolic shift from primary growth to secondary metabolite biosynthesis, likely associated with nutrient limitation and oxidative stress induction. Error bars represent standard deviations from three independent replicates (n = 3).

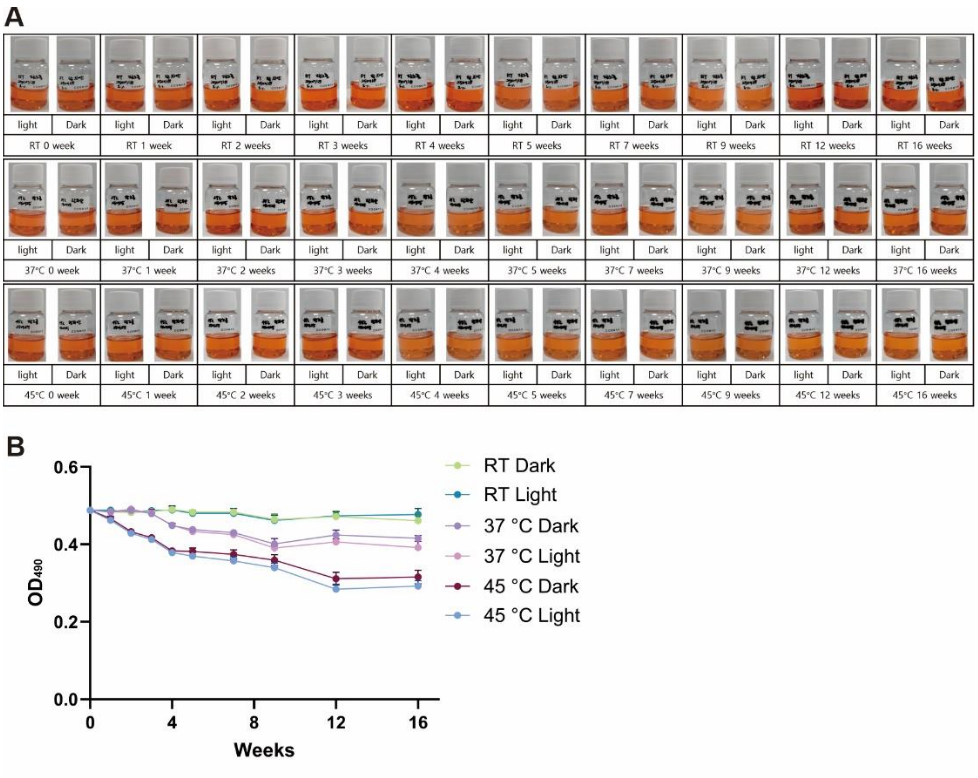

Figure S5. Stability of *R. evergladensis* CXCN-6 extracts under different storage conditions. (A) The color stability of the CXCN-6 extract was evaluated under three temperature conditions (room temperature, 37 °C, and 45 °C) over a period of three months under both light-exposed and dark conditions. No significant change in pigment intensity or hue was observed across treatments, indicating excellent thermal and photostability of the extract. The reddish-orange coloration, primarily attributed to torularhodin, remained visually and spectroscopically stable (absorbance at 490 nm) throughout the storage period, suggesting that the carotenoid composition of CXCN-6 extract exhibits high resistance to oxidative degradation and thermal denaturation. (B) Quantitative stability analysis of CXCN-6 extracts based on absorbance at 490 nm over a 16-week storage period. The extract retained more than 95% of its initial color intensity at 25 °C, indicating outstanding stability. At 37 °C, pigment retention remained above 80%, showing only moderate degradation. A more pronounced decrease (~40% loss) occurred at 45 °C, yet the extract maintained substantial residual pigmentation. Notably, light exposure had no measurable impact on degradation trends, demonstrating that temperature—rather than photochemical reaction—is the

dominant factor affecting pigment stability in the CXCN-6 extract.

Table S1. Assembly Statistics of the sequencing data.

Category	Property
Total Reads	46,353,490
Filtered Reads	45,851,564
Counts of scaffold sequences	18
Length of scaffold sequences	20,719,578
N50 (bp)	1,396,080
Total Assembly Length (Mbp)	20.72
GC Content	62.2%

Table S2. Gene Prediction Statistics of CXCN-6.

Category	Property
Total Genes Length (bp)	14,525,072
Total Genes Num	6,389
Average Gene Length (bp)	2,273
Genes Percentage of Genome (%)	70.0
Total Exons Length (bp)	10,682,549
Total Exons Num.	38,987
Average Exons Length (bp)	274
Average Exons Per Gene	6.0
Total CDSs Length (bp)	10,666,787
Average CDS Length (bp)	1,669
CDSs Percentage of Genome (%)	51.0
